# The expression of nicotinic acetylcholine receptor subunits and their associations with local immune cells and prognosis in oral squamous cell carcinoma

**DOI:** 10.1002/cam4.6482

**Published:** 2023-09-01

**Authors:** Chi‐Maw Lin, Long‐Wei Lin, Tseng‐Cheng Chen, Yi‐Ling Ye, Bor‐Luen Chiang

**Affiliations:** ^1^ Department of Otolaryngology National Taiwan University Hospital, Yun‐Lin Branch Taipei Taiwan; ^2^ Graduate Institute of Clinical Medicine, College of Medicine National Taiwan University Taipei Taiwan; ^3^ Department of Pathology National Taiwan University Hospital, Yun‐Lin Branch Taipei Taiwan; ^4^ Department of Otolaryngology National Taiwan University Hospital and National Taiwan University, College of Medicine Taipei Taiwan; ^5^ Department of Biotechnology National Formosa University Huwei Taiwan

**Keywords:** epithelial–mesenchymal transition (EMT), immune responses, nicotinic acetylcholine receptor (nAChR), oral squamous cell carcinoma (OSCC), smoking

## Abstract

**Background:**

Nicotinic acetylcholine receptors (nAChRs) are ligand‐gated ion channels that may be responsible for cancer cell proliferation, epithelial–mesenchymal transition (EMT), and immune regulation. However, little is known about the associations of different nAChR subunits with tumor microenvironment in oral squamous cell carcinoma (OSCC).

**Methods:**

We retrospectively reviewed pathology samples from 75 OSCC patients by immunohistochemistry. In addition, a cohort of 307 OSCC patients in The Cancer Genome Atlas was analyzed.

**Results:**

Subunit α1 was specific to peri‐OSCC skeletal muscle. Increased α1 was associated with increased CD44 (cancer stem cells), increased CD3 and 8 (T cells), increased CD56 and 16 (natural killer cells), a decreased T stage, and an increased N stage. Increased α3 was associated with increased CD56 and 16. Increased α5 was associated with decreased CD3, 8, and 56, a decreased T stage, an increased N stage, worse survival, and decreased epithelial features. Increased α7 was associated with increased CD3, 8, 56, and 16, decreased tumor/peritumor ratios of CD3, 8, and 56 immune cells, and increased epithelial features. Increased local immune cells were associated with a better prognosis.

**Conclusions:**

α5 is the only subunit associated with decreased local immune cells and worse survival, while α1, α3, and α7 are associated with increased local immune cells in OSCC. α5 and α7 are correlated with different EMT states to be mesenchymal‐like and epithelial‐like OSCC, respectively. Protein expression data of the nAChR subunits, complementary to gene expression data, could provide meaningful information regarding the EMT status of OSCC associated with immune responses and prognosis.

## Graphical abstract

Increased local immune cells were associated with a better prognosis in oral squamous cell carcinoma (OSCC). Nicotinic acetylcholine receptor (nAChR) subunit α5 is the only subunit associated with decreased local immune cells and worse survival, while α1, α3, and α7 are associated with increased local immune cells. α5 and α7 are correlated with different epithelial–mesenchymal transition (EMT) states to be mesenchymal‐like and epithelial‐like OSCC, respectively.

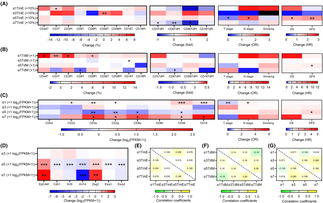

## 1 INTRODUCTION

Oral squamous cell carcinoma (OSCC) is a major subtype of head and neck cancer (HNC) and is known to be the sixth most common cancer worldwide.^1^, ^2^, ^3^, ^4^ Tobacco smoking has been recognized as a main etiological factor responsible for the development and progression of OSCC.^1^, ^5^, ^6^, ^7^, ^8^ Among more than 5000 different substances in tobacco smoke, nicotine is the major ingredient responsible for smoking addiction.^3^, ^5^, ^9^ Moreover, nicotine can be carcinogenic and tumor‐promoting due to its ability to damage the genome, facilitate cancer cell growth, and protect mutated cells from apoptosis.^9^, ^10^, ^11^ These effects mostly act through the binding of nicotine to its primary receptor, which is called the “nicotinic acetylcholine receptor (nAChR)”.^6^, ^9^ Activated nAChRs can modify dopamine neurotransmission in the central nervous system, which is related to tobacco dependence, induce several proliferation‐associated pathways to promote tumor growth, and network with epithelial growth factor receptors to enhance mitogenic effects.^12^, ^13^, ^14^

nAChRs are transmembrane ligand‐gated ion channels formed by five subunits to create a pentameric protein.^9^, ^11^, ^15^, ^16^ There are two types of nAChRs. One type called the “muscle” type is composed of heteromeric combinations of two α1, β1, γ/ε, and δ subunits and is mainly expressed on muscular cells.^9^ The other type, which is called the “neuronal” type, is composed of homomeric α7 or α9 subunits or heteromeric combinations of α2‐6 and β2‐4 subunits and was originally thought to be expressed exclusively on neurons.^9^, ^16^, ^17^ Subsequent studies showed that neuronal nAChRs can also be found in nonneuronal tissues and cells, such as the epithelium, endothelium, several types of cancers, and immune cells.^13^, ^16^, ^18^, ^19^ Previous research demonstrated that the nAChR α3, α5, α7, α9, β2, and β4 subunits are expressed on oral keratinocytes, and that the α1, α3, α5, and α7 subunits are expressed on normal and malignant tissues in the upper aerodigestive tract.^20^, ^21^

In the tumor microenvironment (TME), local immune cells continuously interact with cancer cells, influencing their proliferation, epithelial–mesenchymal transition (EMT), and metastasis.^22^, ^23^, ^24^ CD3^+^CD8^+^ cytotoxic T cells (CTLs) are the main lymphocytes directly fighting differentiated tumors, while CD16^+++^CD56^+^ cytotoxic natural killer (NK) cells and CD16^+/‐^CD56^++^ split‐anergized NK cells are the main immune cells targeting poorly differentiated tumors/cancer stem cells (CSCs).^25^ Existing studies have indicated that a low density of stromal tumor‐infiltrating lymphocytes (TILs) is associated with worse survival in HNC, while a higher infiltration of CTLs and activated NK cells are associated with better survival.^26^, ^27^ In addition, CD44, which can be expressed on normal stem cells and CSCs, is the most frequently used CSC marker in HNC.^28^, ^29^ Various epithelial markers including epithelial cell adhesion molecule (EpCAM), cadherin 1 (Cdh1), keratin (Krt) 5/14, desmoglein 2 (Dsg2), and epithelial splicing regulatory protein 1/2 (Esrp1/2) have been adopted to depict the EMT states of cancers.^30^

It has been found that tobacco smoking and chronic nicotine exposure can either exacerbate pathogenic immune responses or attenuate defensive immunity.^31^, ^32^ nAChRs, as the primary receptors of nicotine, may also be responsible for cancer cell proliferation, EMT, and immune regulation. However, limited information is known about the impacts of different nAChR subunits on TME in OSCC. Therefore, the aims of this study are to explore the expression of the nAChR α1, α3, α5, and α7 subunits in OSCC and clarify their relationships with local immune cells, EMT states, and prognosis.

## 2 METHODS

### 2.1 Patient collections

We retrospectively enrolled OSCC patients diagnosed from January 2014 to March 2016 at National Taiwan University Hospital, Yun‐Lin Branch. Formalin‐fixed, paraffin‐embedded (FFPE) pathology samples of primary tumors were retrieved, and the patients' survival was recorded until July 2021. The corresponding codes of International Classification of Diseases, Tenth Revision (ICD‐10) of these OSCC patients are C00, C02‐C06, and C14.The exclusion criteria were patients with a history of cancer other than HNC, patients who did not have medical records concerning their smoking statuses, and immunocompromised patients who received long‐term steroid treatment. The TNM (tumor, node, and metastases) status of OSCC was classified according to the 2018 criteria of the American Joint Committee on Cancer (AJCC).^33^

To validate the results of our series and incorporate more gene expression data of OSCC patients into our research, we further utilized the publicly available The Cancer Genome Atlas database (TCGA), which provides numerous and precise RNA‐sequencing data of the cancer specimens. The clinical characteristics and gene expression data of OSCC patients were identified and analyzed among TCGA HNC cohort. The RNA expression data were measured by fragments per kilobase of transcript per million mapped reads (FPKM). The nAChR α1, α3, α5, and α7 subunits are coded by the genes CHRNA1, 3, 5, and 7 (Cholinergic Receptor Nicotinic Alpha 1, 3, 5, and 7 Subunit), CD56 is coded by the NCAM1 (Neural Cell Adhesion Molecule 1) gene, and CD16 is coded by the FCGR3A (Fc Fragment of IgG Receptor IIIa) gene. There are 3 different coding genes (CD3d, 3e, and 3 g) for CD3 and 2 different coding genes (CD8a and 8b) for CD8.

Those who smoked less than 100 cigarettes in their lifetime and those who were reformed smokers for more than 15 years were regarded as nonsmokers, while the others were regarded as smokers in both our series and TCGA.^34^, ^35^

### 2.2 Immunohistochemistry (IHC)

The IHC of the nAChR α1, α3, α5, and α7 subunits and CD44, CD3, CD8, CD56, and CD16 was performed using FFPE tumor sections. The rehydration and antigen retrieval of the FFPE sections were performed according to a standard protocol.^36^, ^37^, ^38^ Diaminobenzidine tetrachloride (DAB) chromagen was used to elucidate the antibody–antigen complex. The detailed information of the primary antibodies is as follows: nAChR α1 (PA5‐84868; Thermo Fisher Scientific); nAChR α3 (ab183097; Abcam); nAChR α5 (PA5‐79046; Thermo Fisher Scientific); nAChR α7 (ab216485; Abcam); CD44 (MA5‐13890; Thermo Fisher Scientific); CD3 (MA1‐90582; Thermo Fisher Scientific); CD8 (MA5‐14548; Thermo Fisher Scientific); CD56 (MA5‐11563; Thermo Fisher Scientific); and CD16 (ab183354; Abcam).

The open‐source program ImageJ (version 1.53k) with the plugin IHC‐toolbox was employed for the quantitative analysis of the IHC images.^36^, ^38^ We used the “H‐DAB” color detection model to isolate the color of DAB and converted the image format to the 8‐bite grayscale. After several pretests, we set the most adequate detection threshold at 170 (for the nAChR subunits and CD44) and 150 (for CD3, 8, 56, and 16) and measured the percentages of the stained areas less than the threshold (gray value 0: black; gray value 255: white) as representatives of the IHC expression levels. The percentage of intra‐tumoral tumor‐infiltrating lymphocytes (TILs) and percentage of stromal (peritumor) TILs were measured by the expression levels of CD3, 8, 56, and 16.^39^, ^40^

### 2.3 Statistical analysis

The statistical analyses and graphical representations of the comparative studies were performed using Stata (version 13) and GraphPad Prism (version 9) software. Wilcoxon matched pairs signed rank test and Friedman pairwise test with Dunn's multiple comparisons test were used to compare the expression levels of all markers. To explore the relationships between nAChRs and other CD markers, we used multiple linear regression analyses of the data in our series and TCGA. Logistic regression and ordinal logistic regression analyses were applied to depict the relationships between nAChRs and smoking and the relationships between nAChRs and the T or N stage. A Cox proportional hazards regression was used for the survival analysis. For TCGA, we transformed the normalized RNA‐sequencing count data from FPKM to log_2_(FPKM+1) to fit the hypothesis of a normal distribution in the regression models. Furthermore, Spearman's rank‐order correlation coefficient (R) was used to delineate the relationships between different nAChR subunits. Finally, Youden's index was adopted to determine the optimal cut‐off points for the expression levels of the α5 nAChR subunit, and Kaplan–Meier survival analyses were plotted and examined using the log‐rank test and hazard ratio (HR).

## 3 RESULTS

### 3.1 Patient demographics

In total, 75 eligible OSCC patients were enrolled in our series, and a cohort of 307 OSCC patients was found among 528 HNC patients in the TCGA database. The clinical characteristics of all patients are listed in Table 1, and there were some differences between our series and TCGA. First, 90.67% of the patients were male in our series, while only 67.43% of the patients were male in TCGA. Second, the buccal mucosa and gum were the most common (54.67%) in our series, while the tongue and mouth floor were the most common (60.26%) in TCGA. Third, the newest AJCC 8th edition was used in our series, while the old AJCC 5th, 6th, and 7th editions were used in TCGA. Fourth, T1 and T2 stages were the most common (65.33%) in our series, while T3 and T4 stages were the most common (57.65%) in TCGA. Fifth, only one patient (1.33%) had G3 (Poorly differentiated) histopathological grading in our series, while 63 patients (20.52%) had G3 grading in TCGA.

**TABLE 1:** Clinical characteristics of patients with oral squamous cell carcinoma.

	Our series (*n* = 75)	TCGA (*n* = 307)
Age (mean [standard deviation]) (years)	60.47 (10.28)	61.8 (13.08)
Sex (male)	68/75 (90.67%)	207/307 (67.43%)
Smoking	51/75 (68%)	166/307 (54.07%)
Tumor sites		
Tongue, Mouth floor	23/75 (30.67%)	185/307 (60.26%)
Buccal mucosa, Gum	41/75 (54.67%)	40/307 (13.03%)
Lip, Hard palate	11/75 (14.67%)	10/307 (3.26%)
Oral cavity (unspecified)	0/75 (0%)	72/307 (23.45%)
T stage	AJCC 8th	AJCC ≤7th
T1, 2	49/75 (65.33%)	130/307 (42.35%)
T3, 4	26/75 (34.67%)	177/307 (57.65%)
N stage	AJCC 8th	AJCC ≤7th
N0, 1	58/75 (77.33%)	199/307 (64.82%)
N2, 3	17/75 (22.67%)	105/307 (34.2%)
Unknown	0/75 (0%)	3/307 (0.98%)
Histopathological grading		
G1 (Well differentiated)	18/75 (24%)	48/307 (15.64%)
G2 (Moderately differentiated)	56/75 (74.67%)	192/307 (62.54%)
G3 (Poorly differentiated)	1/75 (1.33%)	63/307 (20.52%)
Unknown	0/75 (0%)	4/307 (1.3%)
Treatment		
OP only (or no adjuvant therapy record)	24/75 (32%)	119/307 (38.76%)
OP + adjuvant therapy	51/75 (68%)	188/307 (61.24%)

*Note*: Adjuvant therapy including chemotherapy, radiotherapy, chemoradiotherapy, or targeted therapy.

Abbreviations: AJCC, the American joint committee on cancer; OP, operation; TCGA, the cancer genome atlas.

### 3.2 Expression levels of nAChR subunits and CD markers in IHC staining

**FIGURE 1:**
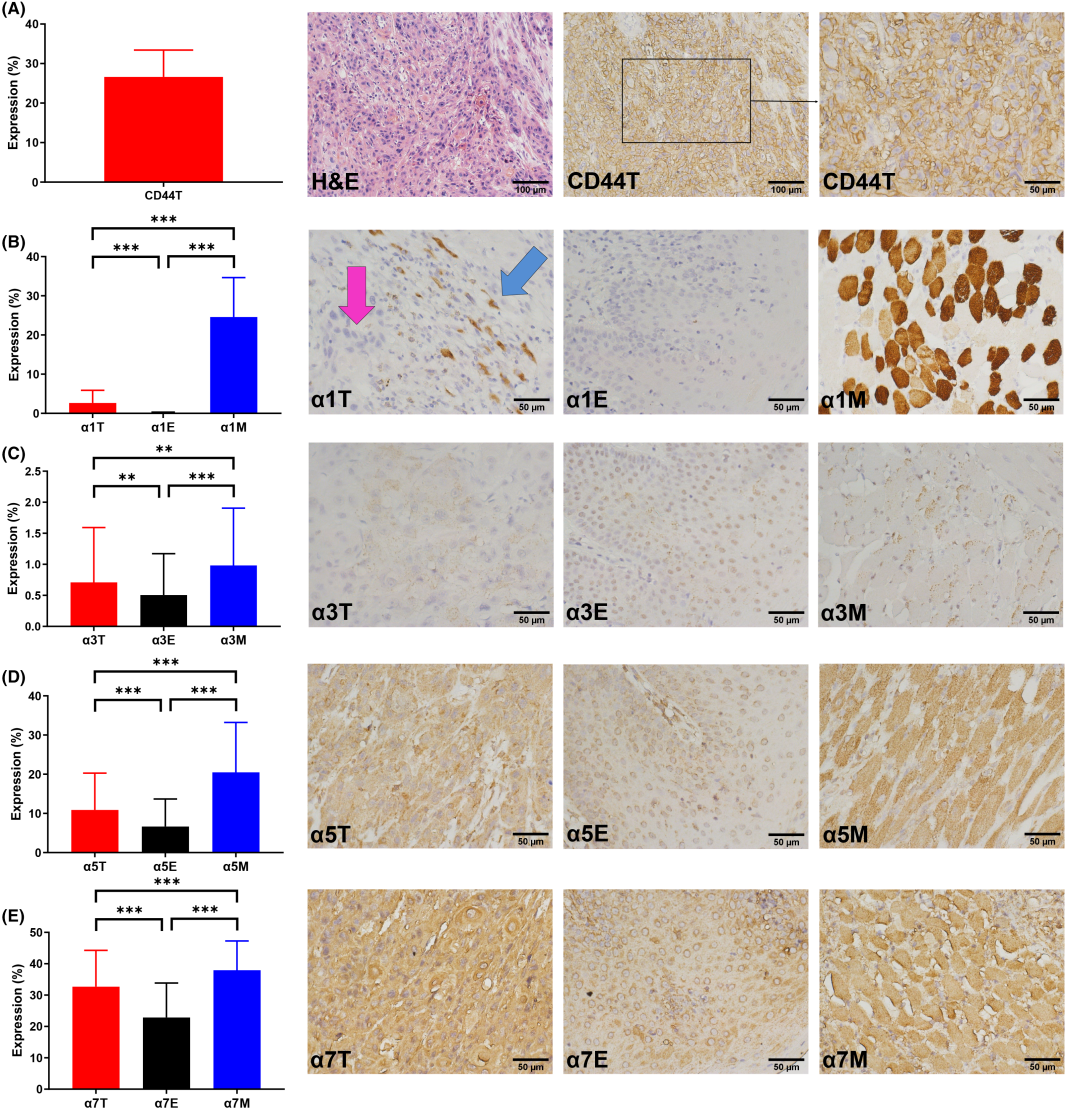
H&E and IHC pathology images of a 50‐year‐old male patient with T3 tongue cancer and the overall expression levels of CD44 (A), the nAChR α1, α3, α5, and α7 subunits (B ‐ E) of 75 OSCC patients in our series. H&E: hematoxylin and eosin; IHC: immunohistochemistry; nAChR: nicotinic acetylcholine receptor; T, tumor; E, epithelium; M, muscle; red arrow: tumor tissue; blue arrow: peritumor skeletal muscle; *p < 0.05, **p < 0.01, ***p < 0.001.

**FIGURE 2:**
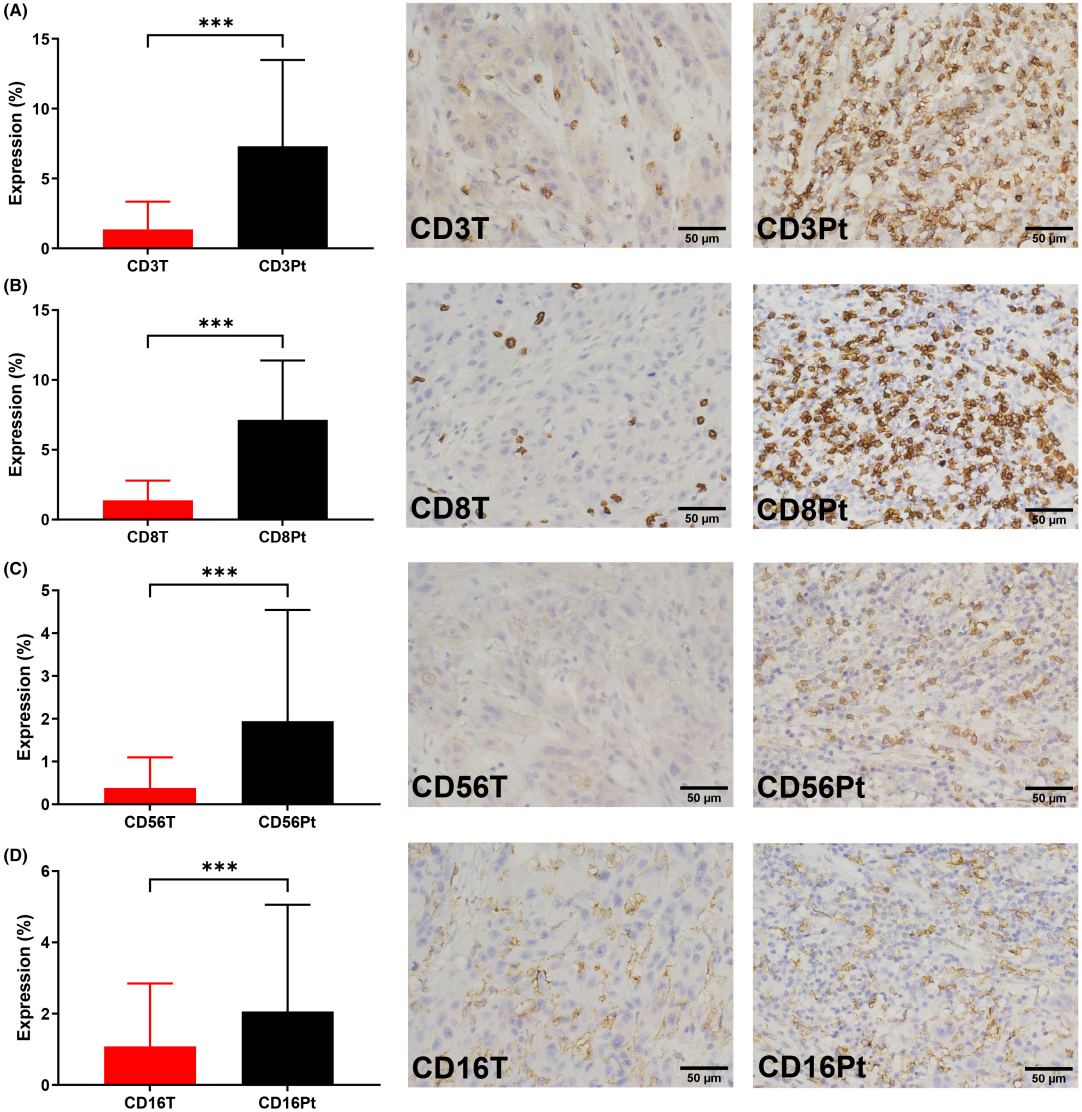
H&E and IHC pathology images of a 50‐year‐old male patient with T3 tongue cancer and the overall expression levels of CD3 (A), CD8 (B), CD56 (C), and CD16 (D) of 75 OSCC patients in our series. H&E: hematoxylin and eosin; IHC: immunohistochemistry; T, tumor; Pt, peritumor; *p < 0.05, **p < 0.01, ***p < 0.001.

The IHC staining statuses of the nAchRs were recorded in three different sites, including the tumor (T), epithelium (E) and muscle (M), while the staining statuses of the T cell markers (CD3 and 8) and NK cell markers (CD56 and 16) were recorded in two different sites, including the tumor (T) and peritumor (Pt). Under the largest magnification power (400×) of our microscope, we chose 3–6 different fields to calculate the mean IHC staining extent and intensity. Figures 1 and 2 show examples of the staining pictures and the expression levels of all markers. There were seven patients whose pathology samples did not contain muscular tissues.

**FIGURE 3:**
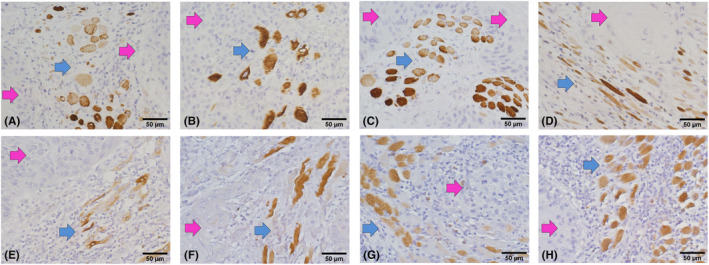
IHC pathology pictures of the nAChR α1 subunit in different OSCC patients (A ‐ H). IHC: immunohistochemistry; OSCC: oral squamous cell carcinoma; nAChR: nicotinic acetylcholine receptor; red arrow: tumor tissue; blue arrow: peritumor skeletal muscle.

Upon a close examination of the pathological findings, it was noted that the IHC staining of the nAChR α1 subunit was highly specific to the skeletal muscle, while OSCC hardly expressed α1. As shown in Figure 3, only the peritumor skeletal muscle could express α1. Therefore, the expression levels of α1 in the tumor and peritumor sites were calculated together in α1T.

The mean expression levels of α1, α3, α5, and α7 were all the strongest in the muscle, followed by the tumor and finally the epithelium (Figure 1). The mean expression levels of CD3, 8, 56, and 16 in the peritumor were all significantly stronger than those in the tumor site (Figure 2).

To calibrate the expression levels of the nAChR subunits for further comparative analyses, we introduced two extra parameters. The first parameter, which was named “TmE” (“tumor minus epithelium”), regards the expression level in the epithelium as the background. The second parameter, which was named “TdM” (“tumor divided by muscle”) regards the expression level in the muscle as a reference. In addition, a new parameter named “TdPt” (“tumor divided by peritumor”) was introduced for the immune cell markers (CD3, 8, 56, and 16).

### 3.3 Comparisons on the expression levels of the markers in our series and TCGA

**FIGURE 4:**
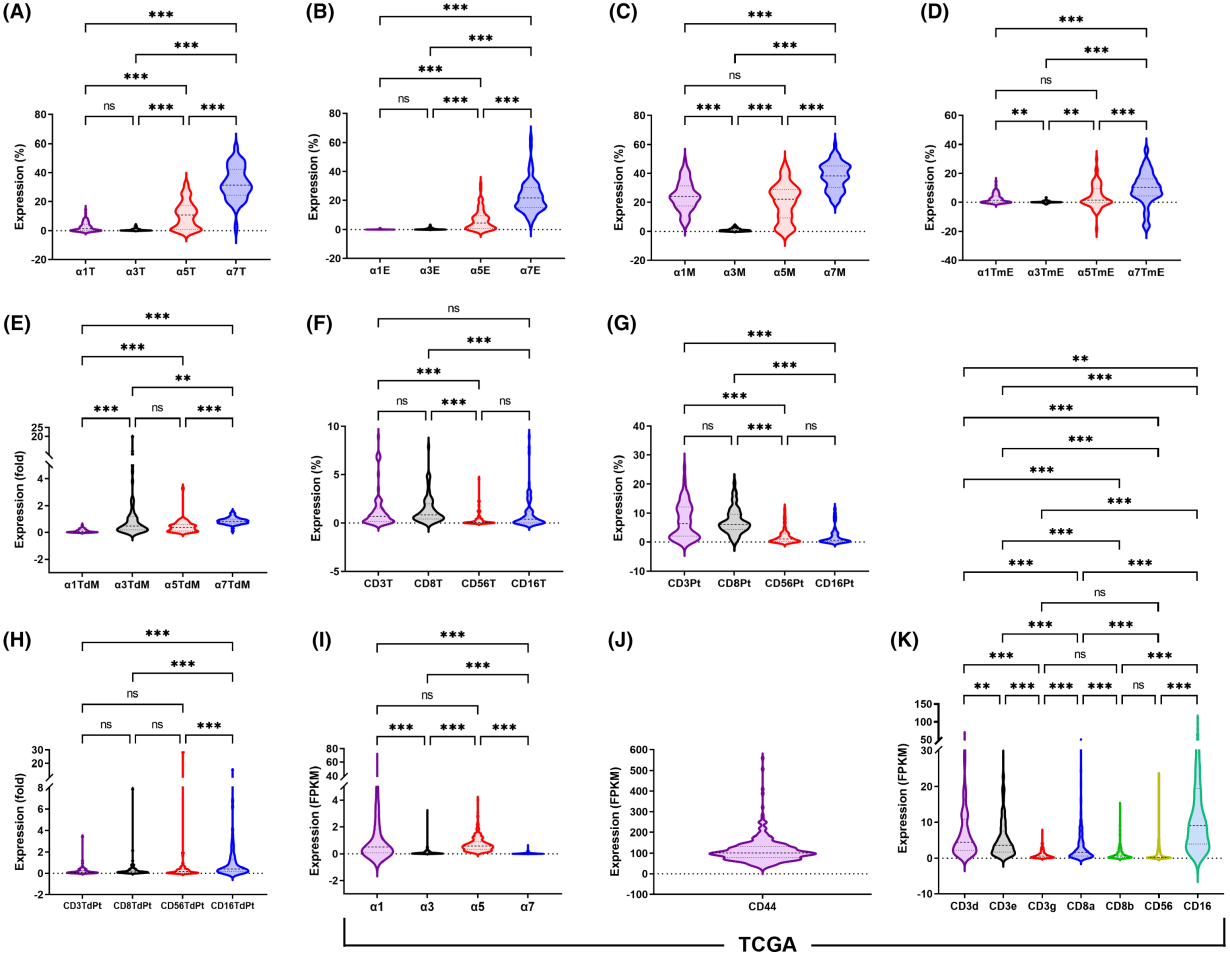
Comparisons of all markers in our series (A – H) and TCGA (I – K). A – E: nAChR in T, E, M, TmE, and TdM; F – H: CD markers in T, Pt, and TdPt; TCGA: The Cancer Genome Atlas; nAChR: nicotinic acetylcholine receptor; T, tumor; E, epithelium; M, muscle; TmE, tumor minus epithelium; TdM, tumor divided by muscle; Pt, peritumor; TdPt, tumor divided by peritumor; *p < 0.05, **p < 0.01, ***p < 0.001.

In TCGA, the expression levels of α1 and α5 were the strongest, followed by α3 and α7 (Figure 4I). However, in our series, the expression level of α7 was the strongest in all 3 sites (including the T, E, and M), followed by α5 in all 3 sites or α1 in muscle and, finally, by α3 in all 3 sites or α1 in the tumor and epithelium **(**Figure 4A–C). This contradiction indicated that the IHC staining of α7 may have high background signals. This pitfall could be minimized by calibrations using TmE and TdM (Figure 4D, E). The high expression level of α1 in TCGA should be mainly derived from the peritumor skeletal muscle.

In TCGA, the gene expression of CD3g, 8b, and 56 was significantly weaker than that of the other CD markers (Figure 4K). In our series, the IHC expression levels of CD56 were significantly weaker than those of CD3 and 8 in both the tumor and peritumor sites (Figure 4F, G).

### 3.4 Multiple regression analyses of the nAChR α1, α3, α5, and α7 subunits in our series

**FIGURE 5:**
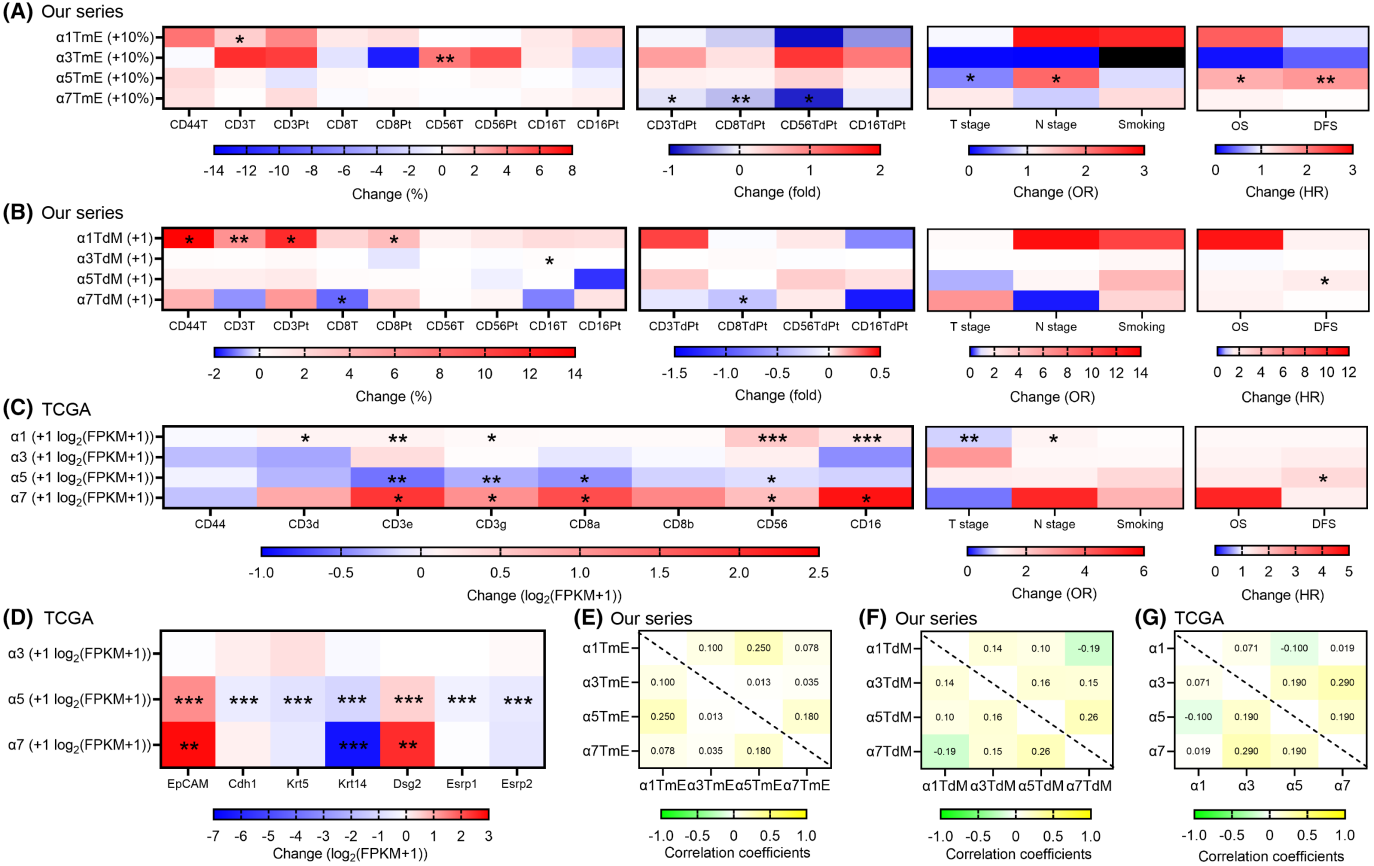
Multiple regression analyses (A – D) and correlation coefficients (E – G). A: nAChR α1, α3, α5, and α7TmE in our series (*n* = 75); B: nAChR α1, α3, α5, and α7TdM in our series (*n* = 68): C: nAChR α1, α3, α5, and α7 subunits in TCGA; D: nAChR α3, α5, and α7 subunits for epithelial markers in TCGA; E: correlation coefficients between nAChR α1, α3, α5, and α7TmE; F: correlation coefficients between nAChR α1, α3, α5, and α7TdM; G: correlation coefficients between nAChR α1, α3, α5, and α7 subunits in TCGA; Black color: out of range; nAChR, nicotinic acetylcholine receptor; T, tumor; TmE, tumor minus epithelium; TdM, tumor divided by muscle; Pt, peritumor; TdPt, tumor divided by peritumor; TCGA, The Cancer Genome Atlas; FPKM, fragments per kilobase of transcript per million mapped reads; EpCAM, Epithelial cell adhesion molecule; Cdh, Cadherin; Krt, Keratin; Dsg, Desmoglein; Esrp, Epithelial splicing regulatory protein; OR, odds ratio; HR, hazard ratio; OS, overall survival; DFS, disease‐free survival; *p < 0.05, **p < 0.01, ***p < 0.001. 95% confidence interval is not shown.

After controlling for age and sex, we performed multiple regression analyses of the nAChR subunits by using the parameter set of nAChR α1, α3, α5, and α7 TmE and the set of nAChR α1, α3, α5, and α7 TdM. As shown in Figure 5A,B, and Table S1 and S2, increased α1 was related to increased CD44T, 3 T, 3Pt, and 8 T. Increased α3 was related to increased CD56T and 16 T. Increased α5 was related to a decreased T stage, an increased N stage, worse overall survival (OS), and worse disease‐free survival (DFS). Finally, increased α7 was related to decreased CD3TdPt, 8 T, 8 TdPt, and 56TdPt.

### 3.5 Multiple regression analyses of the nAChR α1, α3, α5, and α7 subunits in TCGA

Figure 5C and Table S3 show the multiple regression analyses of the nAchR α1, α3, α5, and α7 subunits in TCGA after controlling for age and sex. Increased α1 was related to increased CD3d, 3e, 3 g, 56, and 16, a decreased T stage, and an increased N stage. Increased α5 was related to decreased CD3e, 3 g, 8a, and 56 and worse DFS. Increased α7 was related to increased CD3e, 3 g, 8a, 56, and 16. There were no significant associations between smoking and the expression levels of the nAChR subunits in either our data or TCGA.

### 3.6 Relationships between the nAChR subunits and the EMT states

We performed the multiple regression analyses of the nAchR α3, α5, and α7 subunits to the epithelial markers in TCGA after controlling for age and sex. Subunit α1 was excluded because it was seldom expressed on OSCC. As shown in Figure 5D and Table S4, α3 was not related to any expression alteration of the epithelial markers. Increased α5 was related to decreased expression levels of five epithelial markers (Cdh1, Krt5/14, and Esrp1/2) and increased levels of two epithelial markers (EpCAM and Dsg2). Increased α7 was related to increased expression levels of two epithelial markers (EpCAM and Dsg2) and decreased levels of one epithelial marker (Krt14).

### 3.7 Correlations between different nAChR subunits

The correlation coefficients (R) between different nAChR subunits in our series and TCGA are shown in Figure 5E–G. All correlation coefficients were lower than 0.4, indicating that α1, α3, α5, and α7 were weakly correlated to each other.

### 3.8 Kaplan–Meier survival analyses for nAChR subunit α5

**FIGURE 6:**
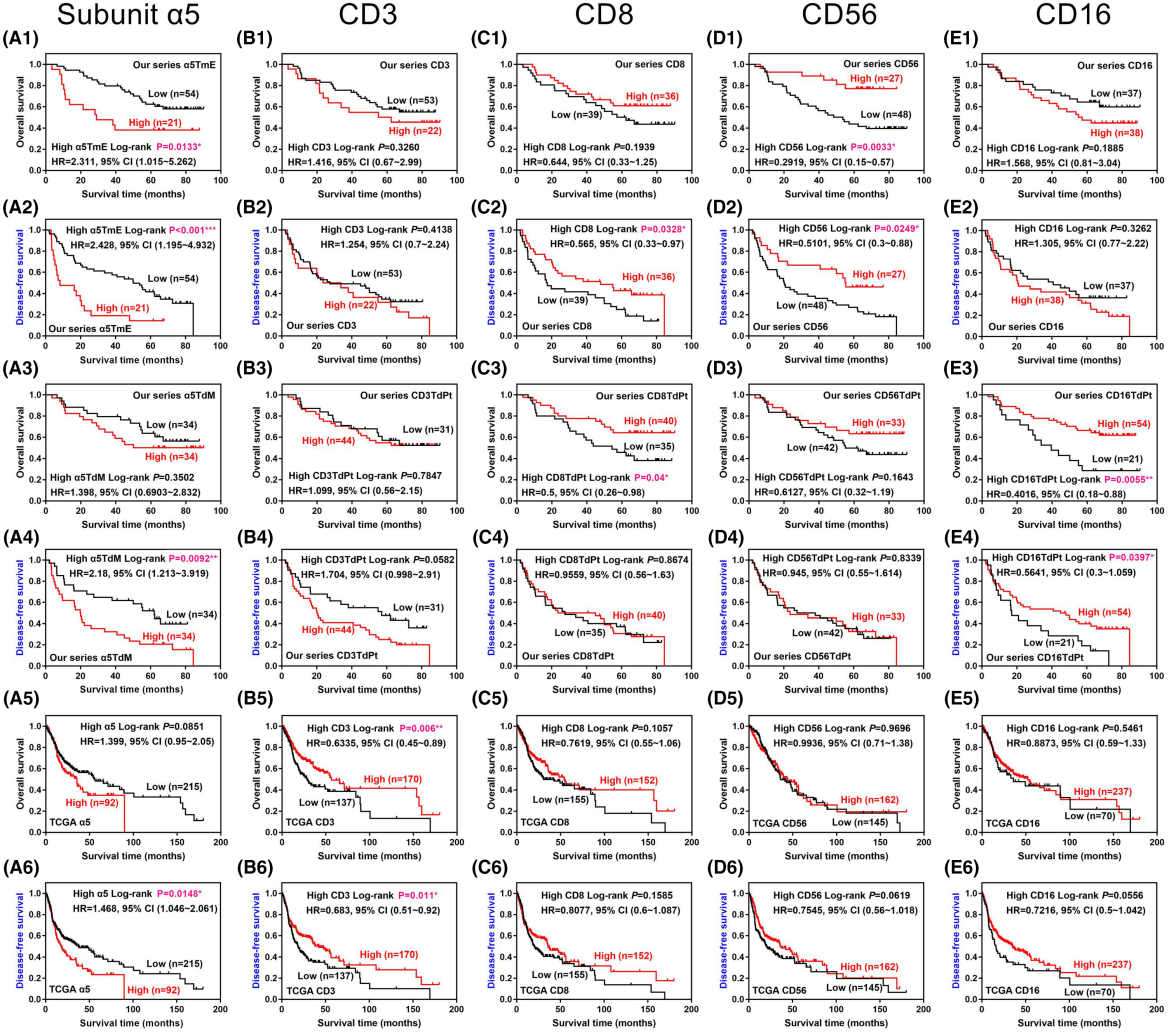
Kaplan–Meier survival plots and log‐rank tests for different expression levels of nAChR subunit α5 (A) and the immune markers (B ‐ E). 1 ~ 2: OS and DFS for α5TmE, CD3, 8, 56, and 16 in our series; 3 ~ 4: OS and DFS for α5TdM, CD3TdPt, 8 TdPt, 56Tdpt, and 16 TdPt in our series; 5 ~ 6: OS and DFS for subunit α5, CD3, 8, 56, and 16 in TCGA; nAChR, nicotinic acetylcholine receptor; TmE, tumor minus epithelium; TdM, tumor divided by muscle; TdPt, tumor divided by peritumor; TCGA, The Cancer Genome Atlas; HR, hazard ratio; OS, overall survival; DFS, disease‐free survival; CI, confidence interval. *p < 0.05, **p < 0.01, ***p < 0.001.

**FIGURE 7:**
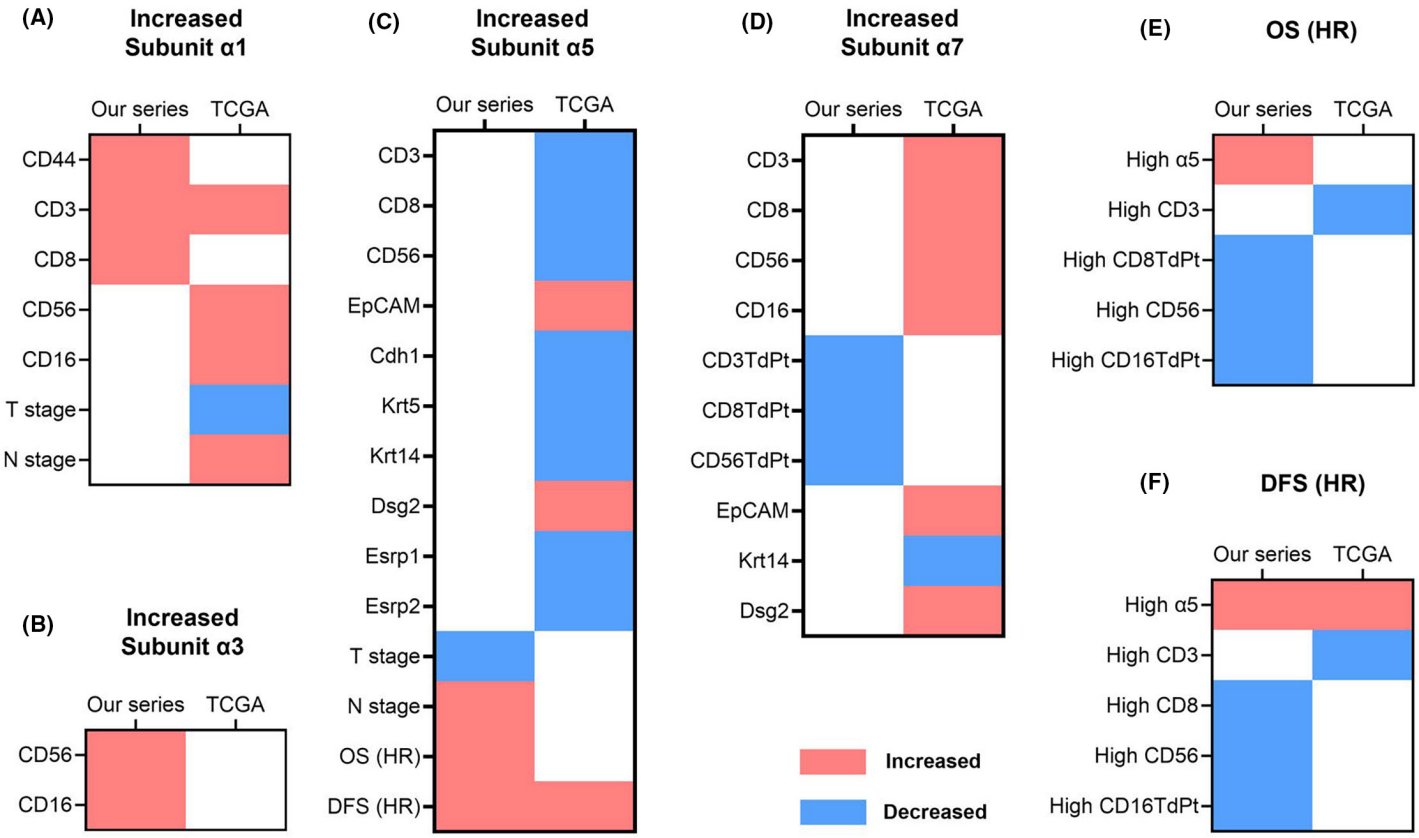
Significant effects for increased nAChR subunit α1, α3, α5, and α7 based on the multiple regression analyses (A ‐ D) and significant HR of survival analyses (E ‐ F) in our series and TCGA. nAChR, nicotinic acetylcholine receptor; TdPt, tumor divided by peritumor; EpCAM, Epithelial cell adhesion molecule; Cdh, Cadherin; Krt, Keratin; Dsg, Desmoglein; Esrp, Epithelial splicing regulatory protein; HR, hazard ratio; OS, overall survival; DFS, disease‐free survival.

The optimal cut‐off points for the expression levels of subunit α5 and the immune markers were determined based on the Youden's index to predict survival or recurrence. In our series, the levels of the immune markers on the tumor and peritumor sites were summed to represent the overall expression. In TCGA, CD3d, 3e, and 3 g were summed to exhibit CD3, and CD8a and 8b were summed to disclose CD8. Figure 6 shows the Kaplan–Meier survival plots. The patients who had high α5TmE levels displayed significant worse OS and DFS than those who had low α5TmE levels. Both high α5TdM and high TCGA α5 levels were significantly related to worse DFS. High CD3 in TCGA and high CD8, CD8TdPt, CD56, and CD16TdPt in our series were significantly associated with a worse prognosis. Figure 7 depicts the summary of the significant findings in our study.

## 4 DISCUSSION

In this study, we used IHC staining to explore the protein expression of the nAChR subunits and adopted TCGA to delineate the gene expression of them in OSCC. The mean IHC expression levels of the nAChR subunits on tumor were stronger than those on tumor‐adjacent benign epithelium. Subunit α1 was specific to peritumor skeletal muscle and seldom expressed on OSCC. A great majority of T and NK cells were located at the peritumor rather than the tumor sites. In IHC staining, the expression level of α7 was the strongest, followed by α5 and α3, while in TCGA, the expression level of α5 was the strongest, followed by α3 and α7. The expressions of α1, α3, α5, and α7 were weakly correlated to each other. There were fewer CD56^+^ NK cells in OSCC TME when compared with CD3^+^ T cells, CD8^+^ T cells, and CD16^+^ NK cells. The nAChR α1, α3, α5, and α7 subunits were significantly associated with the expression alterations of local immune cells, while only α5 had significant impacts on survival in OSCC. Further Kaplan–Meier survival analyses for α5 and immune markers confirmed their prognostic impacts. We also found that subunit α5 and α7 were correlated to decreased and increased epithelial features of OSCC, respectively.

Except for α1, which is specific to skeletal muscle, α3, α5, and α7 are expressed on the oral mucosa and OSCC and could transform to each other according to the different maturation statuses of the cells.^20^ In the oral mucosa, α3 is predominantly expressed on immature basal cells, while α5 and α7 are predominantly expressed on partially and fully differentiated keratinocytes, respectively.^20^, ^41^ These findings are compatible with our study in which subunit α3, α5, and α7 were corresponded to unchanged, decreased, and increased epithelial features, separately. In OSCC, the EMT has been found to be associated with α5‐mediated Stat3‐Jab1/Csn5 and α7‐mediated Akt/YAP/TEAD/BiP signaling,^5^, ^17^ indicating that the transformation of the nAChR subunits between α5 and α7 might be associated with the EMT alteration in OSCC, also compatible with our findings.

Epithelial‐like cancer cells are better surrounded by cancer‐killing immune cells than mesenchymal‐like cancer cells.^24^ Immune attacks from CTLs may convert epithelial‐like cancer cells into mesenchymal‐like cancer cells to exclude CTLs from the TME.^24^ During the EMT process, epithelial‐like cancer cells maintain tight cell–cell adhesion, obscuring the infiltration of immune cells into tumor tissues, whereas mesenchymal‐like cancer cells become more mobile and disseminated, facilitating the infiltration of immune cells into tumor tissues.^24^, ^30^ Altogether, mesenchymal‐like cancer cells might allow more infiltration of immune cells into tumor tissues but decrease the number of immune cells in peritumor regions, hence decreasing the total number of immune cells in the TME because most of the immune cells are at peritumor regions. In contrast, although epithelial‐like cancer cells hinder the infiltration of immune cells into tumor tissues, they increase the number of immune cells in peritumor regions, hence increasing the total number of immune cells in the TME. This scenario is compatible with our study in which increased α5 (decreased epithelial features) was significantly associated with decreased immune cells in the TME, while increased α7 (increased epithelial features) was significantly associated with increased immune cells in the TME and a decreased tumor/peritumor ratio of T and NK cells. Altogether, as shown in the Figure S1, the shifting of the nAChR subunits might reflect/influence the EMT status and thus influence the infiltration of immune cells in OSCC TME.

Increased amounts of peri‐OSCC muscle (α1) indicate that the tumors may be disseminated with smaller tumor sizes (decreased T stage found in TCGA) and may invade deep stromal tissues enriched with blood and lymphatic vessels to facilitate metastasis, resulting in the increased N stage found in TCGA. These deep‐invading OSCCs might be more easily accessible by immune cells from blood and lymphatic vessels and more easily attract immune cells by secreting chemotactic factors, contributing to the increase in T and NK cells in the OSCC TME as shown in our study.

Both epithelial‐like and mesenchymal‐like cancer cells can possess CSC features independent of the EMT.^30^, ^42^ This is compatible with our findings in which α5 and α7 were not significantly associated with the expression alteration of CD44. Although CD44 is related to a worse prognosis in laryngeal and pharyngeal cancer, a previous meta‐analysis study showed that CD44 does not have a significant impact on prognosis in OSCC,^28^ which is also compatible with our study.

In nicotine‐induced lung cancer, a previous study showed that α5‐nAChR can mediate cancer cell proliferation, invasion, and migration and is associated with a reduced survival time.^43^ Another study revealed that chronic stress‐induced acetylcholine elevation could promote lung cancer cell migration and invasion via α5‐nAChR axis.^44^ In HNC, one study showed that α5‐nAChR can significantly predict a worse prognosis, especially in patients receiving radiotherapy, which is strongly associated with the E2F signaling pathway.^8^ In oropharyngeal, hypopharyngeal, and laryngeal cancers, another study showed that advanced cancers are associated with increased transcript levels of the α1 and α5 subunits and decreased transcript levels of the α7 subunit.^21^ In our series, elevated α5 was significantly associated with a decreased T and an increased N stage, which is compatible with the features of mesenchymal‐like cancer cells, that is, weak proliferation capacities but strong invasion and metastasis capacities.^30^ Elevated α5 in OSCC may indicate increased EMT phenotypes and decreased cancer‐killing immune cells in the TME, both of which are associated with a worse prognosis in many cancers.^26^, ^27^, ^30^ On the contrary, though previous study demonstrated that α7‐nAChRs in OSCC cell lines are involved in nicotine‐mediated cell survival and cisplatin resistance,^45^ the α7 expression level was found to have insignificant survival influence in HNC patients,^46^ which is consonant with our study.

In our study, the opposite effects of α5 and α7 on the total number of local immune cells in the OSCC TME are compatible with the literature findings in which smoking can either reduce or increase the number of CTLs and NK cells depending on different pathological conditions.^31^, ^32^ Our study also found that the smoking status was not significantly related to the nAChR subunits, which is compatible with the literature showing that α7‐nAChRs are selectively overexpressed in lung cancer regardless of the smoking status.^9^ One possible explanation is that nAChRs are stimulated by not only nicotine but also autocrine and paracrine acetylcholine from OSCC.^9^

Some limitations of this study should be noted. First, we did not analyze the impacts of the nAChR subunits on the status of human papillomaviruses (HPV), alcohol, and betel nut, which were other major risk factors of HNC apart from smoking. Second, we used CD44 as a single biomarker for CSCs while there are other CSC biomarkers such as NANOG and octamer‐binding transcription factor 4 (Oct‐4). In brief, increased local immune cells were associated with a better prognosis. α5 is the only subunit associated with decreased local immune cells and worse survival, while α1, α3, and α7 are associated with increased local immune cells in OSCC. α5 and α7 are correlated with different EMT states to be mesenchymal‐like and epithelial‐like OSCC, respectively. Protein expression data of the nAChR subunits, complementary to gene expression data, could provide meaningful information regarding the EMT status of OSCC associated with immune responses and prognosis.

## AUTHOR CONTRIBUTIONS

**Chi‐Maw Lin:** Conceptualization (lead); formal analysis (lead); funding acquisition (lead); investigation (lead); project administration (lead); writing – original draft (lead); writing – review and editing (lead). **Long‐Wei Lin:** Investigation (supporting); methodology (supporting). **Tseng‐Cheng Chen:** Supervision (supporting). **Yi‐Ling Ye:** Supervision (equal); writing – original draft (supporting); writing – review and editing (supporting). **Bor‐Luen Chiang:** Supervision (lead); validation (lead); writing – original draft (equal); writing – review and editing (equal).

## FUNDING INFORMATION

This study was financially supported by a project from National Taiwan University Hospital, Yun‐Lin Branch (grant number NTUHYL109.I006).

## CONFLICT OF INTEREST STATEMENT

The authors declare that they have no conflict of interest.

## ETHICS STATEMENT

The Research Ethics Committee of the National Taiwan University hospital approved this study (NTUH IRB‐ 201910107RINC). All methods were performed in accordance with the Declaration of Helsinki.

## CONSENT

Written informed consent was waived by the ethics committee which approved this study.

## Supporting information


**Figure S1.** Proposed representations and impacts of the nAChR α1, α3, α5, and α7 subunits in OSCC correlated to the EMT. nAChR: nicotinic acetylcholine receptor; OSCC: oral squamous cell carcinoma; EMT: epithelial–mesenchymal transition.
**Table S1.** Multiple regression analyses of nAChR α1, α3, α5, and α7TmE (after controlling for age and sex) (*n* = 75)
**Table S2.** Multiple regression analyses of nAChR α1, α3, α5, and α7TdM (after controlling for age and sex) (*n* = 68)
**Table S3.** TCGA multiple regression analyses of the nAChR α1, α3, α5, and α7 subunits (after controlling for age and sex) (*N* = 307)
**Table S4.** TCGA multiple regression analyses of the nAChR α3, α5, and α7 subunits (after controlling for age and sex) (*N* = 307)

## Data Availability

The data generated in this study are available within the article and its supplementary data files.
