# Supplementary material for: The expression of nicotinic acetylcholine receptor subunits and their associations with local immune cells and prognosis in oral squamous cell carcinoma
Source: Cancer Med. 2023 Sep 1;12(18):18918–30. doi: 10.1002/cam4.6482 (PMC10557882; doi:10.1002/cam4.6482)
Supplement: Supplementary file 1 — Figure S1. Proposed representations and impacts of the nAChR α1, α3, α5, and α7 subunits in OSCC correlated to the EMT. nAChR: nicotinic acetylcholine receptor; OSCC: oral squamous cell carcinoma; EMT: epithelial–mesenchymal transition. Table S1. Multiple regression analyses of nAChR α1, α3, α5, and α7TmE (after controlling for age and sex) (n = 75) Table S2. Multiple regression analyses of nAChR α1, α3, α5, and α7TdM (after controlling for age and sex) (n = 68) Table S3. TCGA multiple regression analyses of the nAChR α1, α3, α5, and α7 subunits (after controlling for age and sex) (N = 307) Table S4. TCGA multiple regression analyses of the nAChR α3, α5, and α7 subunits (after controlling for age and sex) (N = 307) [file CAM4-12-18918-s001.pdf]

Contents

Table S1. Multiple regression analyses of nAChR α1, α3, α5, and α7TmE (after controlling for age and sex) (n=75)

Table S2. Multiple regression analyses of nAChR α1, α3, α5, and α7TdM (after controlling for age and sex) (n=68)

Table S3. TCGA multiple regression analyses of the nAChR α1, α3, α5, and α7 subunits (after controlling for age and sex) (N=307)

Table S4. TCGA multiple regression analyses of the nAChR α3, α5, and α7 subunits (after controlling for age and sex) (N=307)

Supplementary Figure S1. Proposed representations and impacts of the nAChR α1, α3, α5, and α7 subunits in OSCC correlated to the EMT. nAChR: nicotinic acetylcholine receptor; OSCC: oral squamous cell carcinoma; EMT: epithelial–mesenchymal transition.

Supplementary Table S1. Multiple regression analyses of nAChR α1, α3, α5, and α7TmE (after controlling for age and sex) (n=75)

	α1TmE (+10%)		α3TmE (+10%)		α5TmE (+10%)		α7TmE (+10%)	
	Effect (95% CI)	P value	Effect (95% CI)	P value	Effect (95% CI)	P value	Effect (95% CI)	P value
Change (%)								
CD44T	4.39 (-0.51~9.29)	0.078	-0.32 (-25.05~24.4)	0.979	1.13 (-0.91~3.16)	0.273	0.89 (-0.49~2.28)	0.201
CD3T	1.51 (0.15~2.87)	0.03	6.55 (-0.32~13.42)	0.061	0.32 (-0.24~0.89)	0.26	0.032 (-0.35~0.42)	0.869
CD3Pt	3.76 (-0.61~8.13)	0.09	6.072 (-15.99~28.13)	0.585	-1.51 (-3.33~0.31)	0.102	1.067 (-0.17~2.30)	0.089
CD8T	0.67 (-0.36~1.70)	0.199	-1.54 (-6.75~3.66)	0.556	0.19 (-0.23~0.62)	0.37	-0.29 (-0.58~0.0047)	0.054
CD8Pt	0.98 (-2.25~4.21)	0.547	-12.51 (-28.82~3.80)	0.13	0.07 (-1.27~1.41)	0.917	0.19 (-0.72~1.10)	0.676
CD56T	-0.072 (-0.56~0.42)	0.771	4.25 (1.78~6.74)	0.001	0.022 (-0.18~0.23)	0.829	-0.12 (-0.26~0.021)	0.095
CD56Pt	-0.17 (-2.15~1.80)	0.861	5.55 (-4.41~15.51)	0.27	0.24 (-0.58~1.061)	0.561	-0.15 (-0.71~0.41)	0.589
CD16T	0.69 (-0.64~2.025)	0.303	0.59 (-6.14~7.31)	0.863	-0.12 (-0.67~0.44)	0.678	0.27 (-0.11~0.64)	0.16
CD16Pt	1.43 (-0.76~3.61)	0.196	-2.58 (-13.60~8.44)	0.642	-0.88 (-1.79~0.025)	0.056	0.48 (-0.14~1.096)	0.124
Change (fold)								
CD3TdPt	-0.042 (-0.35~0.27)	0.79	0.76 (-0.81~2.33)	0.337	0.12 (-0.01~0.25)	0.07	-0.11 (-0.20~-0.026)	0.012
CD8TdPt	-0.20 (-0.89~0.48)	0.554	0.23 (-3.22~3.68)	0.894	0.09 (-0.19~0.37)	0.531	-0.27 (-0.47~ -0.08)	0.006
CD56TdPt	-0.95 (-3.34~1.44)	0.429	1.57 (-10.49~13.63)	0.796	0.33 (-0.67~1.32)	0.514	-0.87 (-1.54~-0.19)	0.012
CD16TdPt	-0.43 (-2.19~1.33)	0.626	1.045 (-7.84~9.93)	0.815	0.11 (-0.62~0.84)	0.767	-0.087 (-0.58~0.41)	0.727
Change (OR)								
T stage	0.97 (0.26~3.67)	0.963	0.023 (0.00~47.49)	0.333	0.52 (0.28~0.97)	0.038	1.16 (0.78~1.73)	0.471
N stage	2.83 (0.59~13.55)	0.194	0.011 (0.00~55.11)	0.299	2.18 (1.14~4.16)	0.019	0.80 (0.50~1.29)	0.361
Smoking	2.70 (0.33~22.25)	0.357	46.56 (0.00~6806703)	0.527	0.86 (0.41~1.80)	0.689	1.28 (0.79~2.077)	0.32
Change (HR)								
OS	2.26 (0.78~6.56)	0.132	0.063 (0.00~105.26)	0.465	1.63 (1.021~2.61)	0.041	1.087 (0.82~1.44)	0.558
DFS	0.90 (0.33~2.49)	0.839	0.37 (0.0034~38.94)	0.674	1.81 (1.25~2.61)	0.002	1.009 (0.81~1.26)	0.935

nAChR, nicotinic acetylcholine receptor; T, tumor; TmE, tumor minus epithelium; Pt, peritumor; TdPt, tumor divided by peritumor; OR, odds ratio; HR, hazard ratio; OS, overall survival; DFS, disease-free survival; CI, confidence interval; Underline, statistically significant (P<0.05).

Supplementary Table S2. Multiple regression analyses of nAChR α1, α3, α5, and α7TdM (after controlling for age and sex) (n=68)

	α1TdM (+1)		α3TdM (+1)		α5TdM (+1)		α7TdM (+1)	
	Effect (95% CI)	P value	Effect (95% CI)	P value	Effect (95% CI)	P value	Effect (95% CI)	P value
Change (%)								
CD44T	13.8 (2.22~25.5)	0.02	0.16 (-0.31~0.63)	0.491	0.91 (-2.6~4.4%)	0.604	4.22 (-2.03~10.5)	0.182
CD3T	5.93 (2.56~9.28)	0.001	0.08 (-0.06~0.21)	0.261	0.91 (-0.1~1.93)	0.077	-0.86 (-2.67~0.95)	0.348
CD3Pt	11.6 (0.42~22.9)	0.042	0.3 (-0.15~0.75)	0.186	1.13 (-2.26~4.5)	0.508	5.64 (-0.41~11.68)	0.067
CD8T	2.3 (0.14~4.46)	0.037	-0.013 (-0.1~0.07)	0.764	0.2 (-0.45~0.85)	0.544	-1.19 (-2.35~-0.02)	0.046
CD8Pt	3.63 (-3.95~11.2)	0.342	-0.21 (-0.51~0.09)	0.172	0.13 (-2.16~2.4)	0.91	2.59 (-1.49~6.67)	0.21
CD56T	0.62 (-0.77~1.99)	0.376	-0.003 (-0.06~0.05)	0.92	0.023 (-0.39~0.44)	0.911	0.076 (-0.67~0.82)	0.838
CD56Pt	1.2 (-3.93~6.33)	0.641	-0.03 (-0.24~0.17)	0.753	-0.12 (-1.67~1.43)	0.877	0.44 (-2.32~3.2)	0.751
CD16T	1.89 (-1.45~5.23)	0.263	0.14 (0.01~0.28)	0.035	0.009 (-1.0~1.01)	0.986	-1 (-2.8~0.8)	0.271
CD16Pt	1.71 (-4.13~7.55)	0.56	0.03 (-0.2~0.27)	0.794	-1.61 (-3.38~0.15)	0.072	1.52 (-1.62~4.67)	0.336
Change (fold)								
CD3TdPt	0.37 (-0.04~0.77)	0.079	0.0006 (-0.02~0.02)	0.941	0.103 (-0.02~0.23)	0.1	-0.143 (-0.36~0.077)	0.198
CD8TdPt	-0.024 (-0.64~0.59)	0.939	0.009 (-0.02~0.03)	0.471	-0.005 (-0.19~0.18)	0.959	-0.356 (-0.69~-0.024)	0.036
CD56TdPt	0.044 (-0.62~0.71)	0.896	-0.0125 (-0.04~0.01)	0.351	0.098 (-0.1~0.299)	0.322	0.04 (-0.317~0.4)	0.822
CD16TdPt	-0.71 (-5.36~3.94)	0.762	-0.002 (-0.19~0.19)	0.987	0.058 (-1.35~1.46)	0.934	-1.36 (-3.86~1.14)	0.282
Change (OR)								
T stage	1.24 (0.04~37.4)	0.9	1.01 (0.89~1.14)	0.843	0.69 (0.27~1.8)	0.455	6.42 (0.82~50.21)	0.076
N stage	13.25 (0.28~626.3)	0.189	1.04 (0.91~1.19)	0.529	1.41 (0.38~5.24)	0.609	0.098 (0.007~1.33)	0.081
Smoking	10.58 (0.06~1825.7)	0.369	1.39 (0.8~2.4)	0.241	4.62 (0.39~54.5)	0.224	3.1 (0.23~41.4)	0.392
Change (HR)								
OS	11.1 (0.85~144.5)	0.066	0.98 (0.87~1.11)	0.772	1.43 (0.74~2.75)	0.283	1.52 (0.35~6.69)	0.58
DFS	1.56 (0.15~16.56)	0.714	1.0007 (0.92~1.09)	0.987	1.796 (1.09~2.95)	0.021	1.05 (0.32~3.44)	0.939

nAChR, nicotinic acetylcholine receptor; T, tumor; TdM, tumor divided by muscle; Pt, peritumor; TdPt, tumor divided by peritumor; OR, odds ratio; HR, hazard ratio; OS, overall survival; DFS, disease-free survival; CI, confidence interval; Underline, statistically significant (P<0.05).

Supplementary Table S3. TCGA multiple regression analyses of the nAChR α1, α3, α5, and α7 subunits (after controlling for age and sex) (N=307)

	α1 (+1 log_2(FPKM+1))		α3 (+1 log_2(FPKM+1))		α5 (+1 log_2(FPKM+1))		α7 (+1 log_2(FPKM+1))	
	Effect (95% CI)	P value	Effect (95% CI)	P value	Effect (95% CI)	P value	Effect (95% CI)	P value
Change (log_2(FPKM+1))								
CD44	-0.024 (-0.07~0.026)	0.348	-0.26 (-0.65~0.12)	0.181	-0.051 (-0.24~0.14)	0.603	-0.24 (-1.19~0.70)	0.613
CD3d	0.11 (0.011~0.20)	0.029	-0.35 (-1.081~0.38)	0.347	-0.29 (-0.65~0.073)	0.118	0.84 (-0.94~2.62)	0.355
CD3e	0.11 (0.029~0.19)	0.008	0.33 (-0.30~0.96)	0.305	-0.55 (-0.86~-0.23)	0.001	1.97 (0.43~3.51)	0.012
CD3g	0.057 (0.007~0.11)	0.025	0.024 (-0.36~0.41)	0.902	-0.32 (-0.51~-0.13)	0.001	1.081 (0.15~2.01)	0.023
CD8a	0.046 (-0.043~0.14)	0.310	-0.098 (-0.79~0.59)	0.779	-0.43 (-0.77~-0.091)	0.013	1.74 (0.067~3.42)	0.042
CD8b	0.043 (-0.020~0.11)	0.179	-0.024 (-0.51~0.46)	0.922	-0.18 (-0.42~0.059)	0.139	1.17 (-0.005~2.34)	0.051
CD56	0.52 (0.49~0.55)	<0.001	0.15 (-0.08~0.39)	0.201	-0.13 (-0.25~-0.013)	0.030	0.63 (0.059~1.21)	0.031
CD16	0.23 (0.13~0.33)	<0.001	-0.45 (-1.23~0.32)	0.252	-0.18 (-0.56~0.21)	0.374	2.32 (0.43~4.22)	0.017
Change (OR)								
T stage	0.82 (0.71~0.95)	0.009	3.02 (0.86~10.65)	0.085	1.32 (0.76~2.28)	0.319	0.46 (0.032~6.39)	0.561
N stage	1.21 (1.04~1.41)	0.013	1.12 (0.36~3.52)	0.844	1.29 (0.73~2.27)	0.376	5.24 (0.29~96.33)	0.265
Smoking	1.053 (0.89~1.25)	0.541	1.11 (0.31~3.97)	0.876	1.75 (0.91~ 3.36)	0.093	2.48 (0.11~58.48)	0.572
Change (HR)								
OS	1.10 (0.99~1.24)	0.087	1.16 (0.46~2.96)	0.754	1.3 (0.83~2.027)	0.246	4.43 (0.45~43.71)	0.202
DFS	1.10 (0.997~1.22)	0.058	1.27 (0.52~3.10)	0.599	1.59 (1.08~2.33)	0.019	1.24 (0.15~10.36)	0.843

TCGA, The Cancer Genome Atlas; nAChR, nicotinic acetylcholine receptor; FPKM, fragments per kilobase of transcript per million mapped reads; OR, odds ratio; HR, hazard ratio; OS, overall survival; DFS, disease-free survival; CI, confidence interval; Underline, statistically significant (P<0.05).

Supplementary Table S4. TCGA multiple regression analyses of the nAChR α3, α5, and α7 subunits (after controlling for age and sex) (N=307)

	α3 (+1 $\log_2$(FPKM+1))		α5 (+1 $\log_2$(FPKM+1))		α7 (+1 $\log_2$(FPKM+1))	
$\log_2$(FPKM+1)	Effect (95% CI)	P value	Effect (95% CI)	P value	Effect (95% CI)	P value
EpCAM	-0.080 (-0.93~0.77)	0.853	1.33 (0.91~1.74)	<0.001	2.88 (0.82~ 4.95)	0.006
Cdh1	0.21 (-0.33~0.74)	0.443	-0.56 (-0.82~-0.29)	<0.001	0.17 (-1.13~1.47)	0.799
Krt5	0.37 (-0.33~1.07)	0.297	-0.81 (-1.16~-0.47)	<0.001	-0.63 (-2.34~1.081)	0.469
Krt14	-0.17 (-1.12~0.77)	0.722	-1.15 (-1.61~-0.68)	<0.001	-6.52 (-8.83~-4.21)	<0.001
Dsg2	-0.0050 (-0.60~0.59)	0.987	0.54 (0.25~0.84)	<0.001	2.48 (1.036~3.93)	0.001
Esrp1	-0.015 (-0.46~0.43)	0.948	-0.40 (-0.62~-0.18)	<0.001	-0.079 (-1.16~1.00)	0.885
Esrp2	0.066 (-0.40~0.53)	0.779	-0.62 (-0.85~-0.39)	<0.001	-0.70 (-1.83~0.43)	0.224

TCGA, The Cancer Genome Atlas; nAChR, nicotinic acetylcholine receptor; FPKM, fragments per kilobase of transcript per million mapped reads; EpCAM, Epithelial cell adhesion molecule; Cdh, Cadherin; Krt, Keratin; Dsg, Desmoglein; Esrp, Epithelial splicing regulatory protein; CI, confidence interval; Underline, statistically significant (P<0.05).

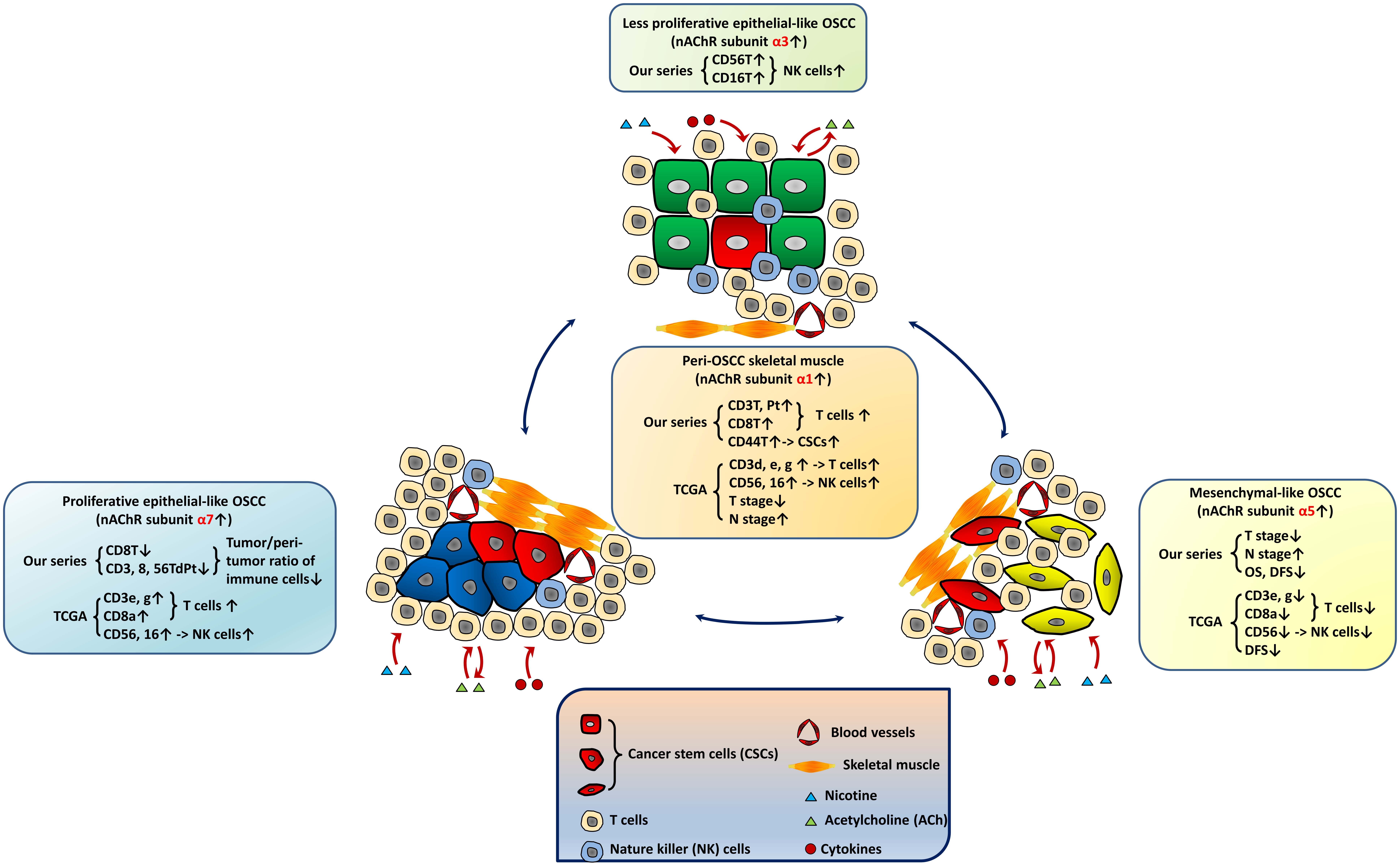

Supplementary Figure S1. Proposed representations and impacts of the nAChR α1, α3, α5, and α7 subunits in OSCC correlated to the EMT. nAChR: nicotinic acetylcholine receptor; OSCC: oral squamous cell carcinoma; EMT: epithelial–mesenchymal transition.
